# Appraising efficiency of OpSite as coolant in drilling of bone

**DOI:** 10.1186/s13018-020-01710-w

**Published:** 2020-05-29

**Authors:** Mohammad Reza Effatparvar, Nima Jamshidi, Alireza Mosavar

**Affiliations:** 1 Department of Biomedical Engineering, Faculty of Engineering, University of Isfahan, Isfahan, Iran; 2 School of Mechanical Engineering, College of Engineering, University of Tehran, Tehran, Iran

**Keywords:** Bone drilling, OpSite, Osteonecrosis, Saline, Temperature variation, Cooling

## Abstract

**Background:**

During drilling of bone, which is common in clinical surgeries, heat generation increases local temperature in the drilling site. Transmission of excessive heat to the surrounding bone tissue can cause thermal osteonecrosis. Consequently, it may lead to failure of implants and fixation screws or delay in healing process. Using cooling is a method for limiting temperature elevation.

**Materials and methods:**

In this study, through comparing three conditions of drilling without cooling, external cooling with normal saline, and external cooling with OpSite spray, the efficiency of OpSite as coolant is studied. In this regard, 2 drill bit diameters, 3 drilling speeds, and 3 drilling feed-rates are considered as drilling variables in the experiments.

**Results:**

For the whole experiments, while cooling with normal saline resulted in lower maximum temperatures than without cooling condition, OpSite had even better results and limited the temperature elevation during drilling of bone efficiently.

**Conclusion:**

OpSite spray, which has lower infection risks than normal saline on one hand and lower maximum temperature rise with all combinations of drilling parameters on the other hand, can be considered in clinical surgeries for cooling applications.

## Introduction

During drilling of bone, which is common in orthopaedic surgeries and prosthodontics [1], plastic deformation of bone chips, friction between the bone and drill bit, and also friction between chips and hole wall cause heat generation in the region [2, 3]. The generated heat increases temperature and can cause thermal osteonecrosis which is death of bone cells (osteocytes) due to thermal overload [4]. Subsequently, it increases risks of failure of implants or delay in healing process. Hence, there are various studies to decrease and control the heat generation. Evaluating different drilling methods, such as ultrasonic-assisted drilling [2] and water jet drilling [5], drilling techniques, like as one step and gradual drilling techniques [6, 7], drilling parameters, including drilling speed, force, and feed-rate [6, 8–12], tool parameters (geometry) [8, 12–14], and cooling conditions, including internal and external cooling methods [6, 8, 15–17] and various coolants [3] are amongst the main efforts in this regard.

In order to control the heat generation in drilling site, while there were positive results for external irrigation in the literature [6, 8, 16, 17], there is a risk of infection for common cooling fluids (water and normal saline) in orthopedic surgery which restricts their usage [3].

To prevent the risk of infection, the aim of this in vitro study was to investigate the efficiency of external cooling with OpSite spray and to compare it to cooling with normal saline. OpSite is a bio-compatible adherent polyurethane film which is waterproof and permeable to water vapor and oxygen [18]. It has clinical applications for providing moist wound environment in superficial wounds and for secondary dressing. According to the aim, various drilling conditions were considered. The experiments were conducted in various combinations of 3 different drilling feed-rates, 3 different drilling speeds, and 2 drill bit diameters.

## Materials and methods

### Bone specimens

For in vitro drilling, bovine Femur diaphysis was bought and employed which is common in orthopaedic animal-experiments with concern to its similar properties to human bone [13, 19]; however, according to the aim of the study in determining efficiency of the coolant in controlling temperature rise, any possible discrepancy in bone properties does not actually hurt deductions. Fresh bones were prepared according to the literature [20]. Although body blood flow acts as coolant during drilling in vivo, it is negligible based on literature [1].

### Experimental parameters

For drilling, a universal mill was employed and orthopaedic drill bits with 2.7 and 3.5 mm diameters were used. Moreover, 3 drilling speeds of 500, 1000, and 1500 rpm and 3 drilling feed-rates of 35, 65, and 85 mm/min were considered. In regard to assessing coolant efficiency, three conditions were investigated for every combination of the mentioned drilling parameters, without cooling, external cooling with normal saline, and external cooling with OpSite spray. Furthermore, to ensure the precision and validity of results, the experiments were repeated twice.

### Investigation of temperature variations

**Fig. 1:**
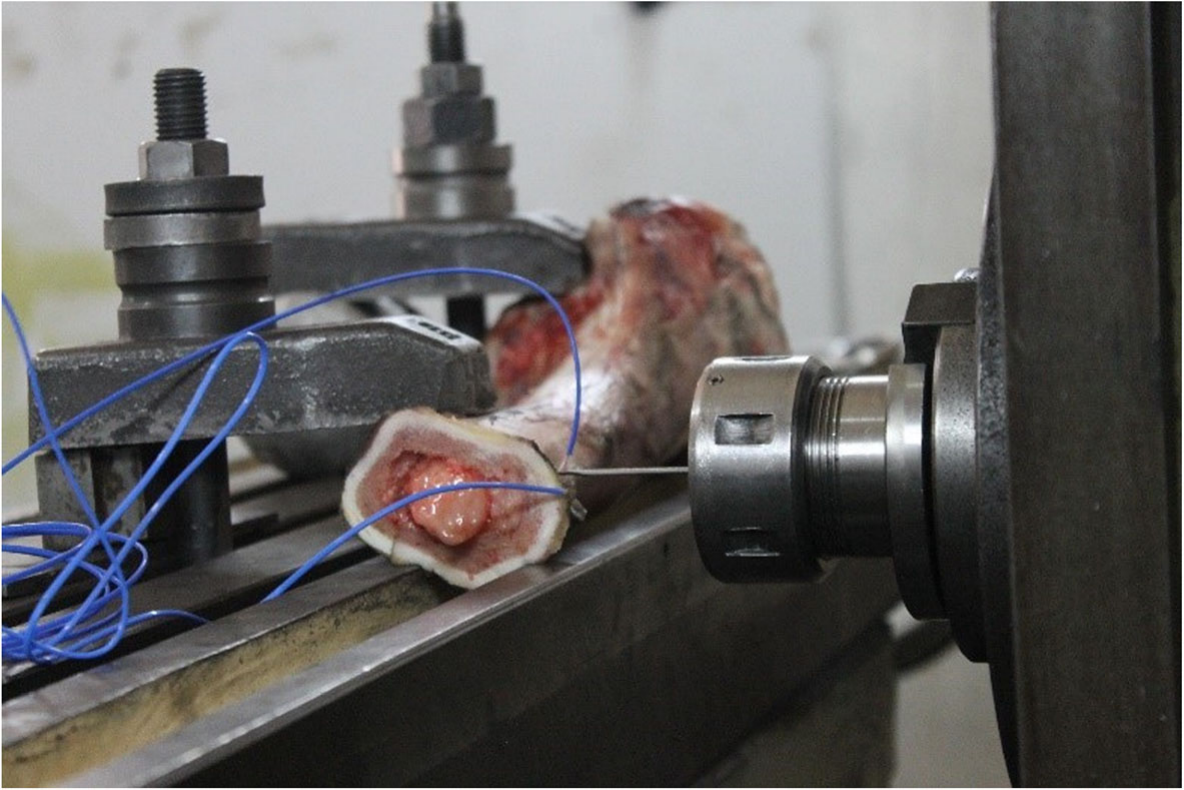
The setup for drilling and temperature measurement of bovine femoral diaphysis. The employed universal drill had options for regulation of drill speed and feed-rate. Also, the maximum bone temperatures during drilling were measured with two thermocouples

In order to record temperature variations during drilling, the temperature is often measured with two methods, either with thermocouples [12, 21] or infrared thermographic camera [6, 14]. In this study, we used type K thermocouples. The distance between drilling site and thermocouple site was 0.5 mm and the depth in which thermocouple was placed in the cortical bone was 3 mm [8]. Two thermocouples were implemented to measure the temperature variations around each drilling hole (Fig. 1) and average record of them was considered as outcome. In addition, the room temperature was 29 °C and the bones warmed up to the same temperature.

## Results

**Fig. 2:**
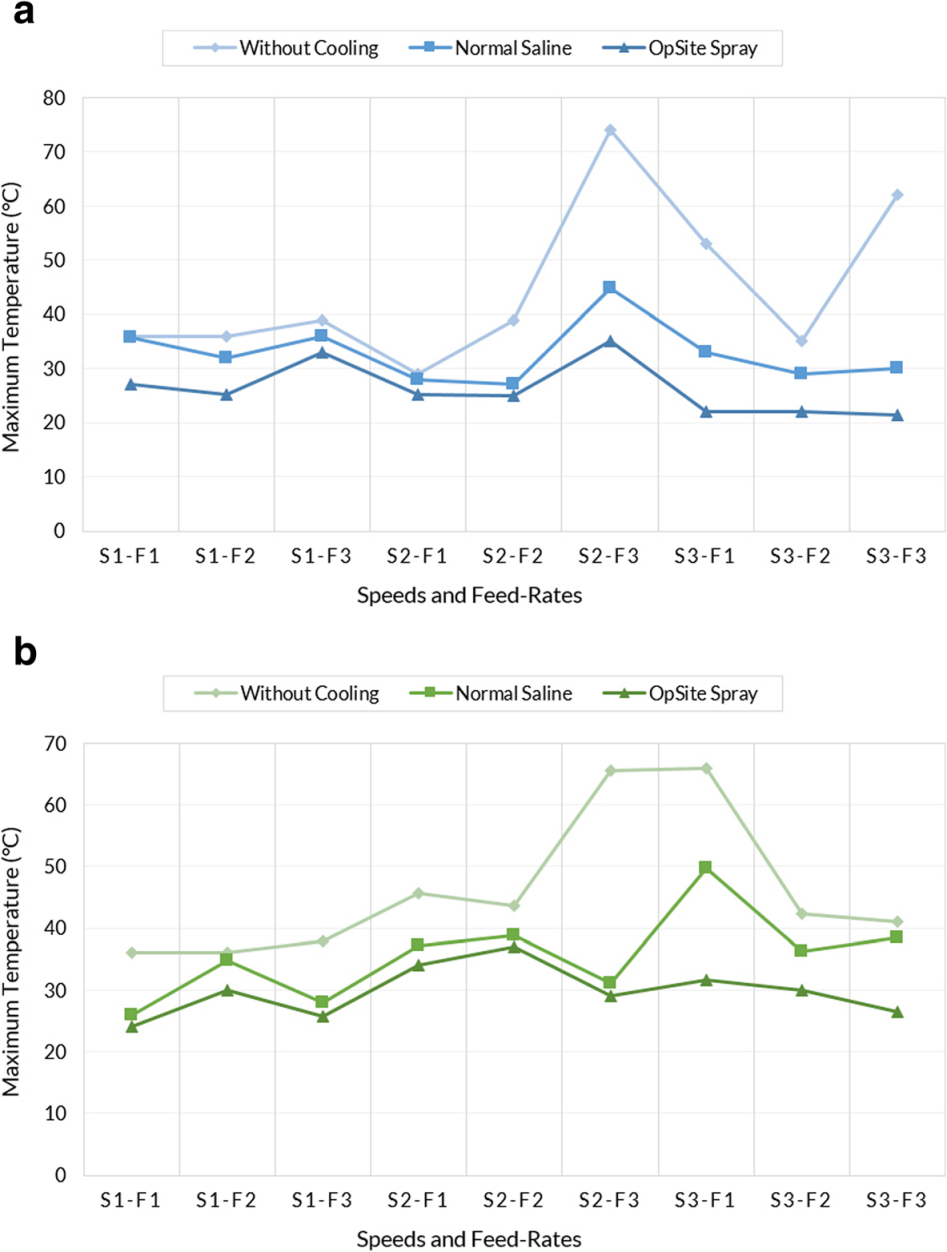
Diagrams of maximum recorded temperature in experiments with **a** drill bit diameter of 2.7 mm, and **b** drill bit diameter of 3.5 mm. In the diagrams the following codes are used. S1: speed of 500 rpm; S2: speed of 1000 rpm; S3: speed of 1500 rpm; F1: feed-rate of 35 mm/min; F2: feed-rate of 65 mm/min; F3: feed-rate of 85 mm/min. Therefore, for example, first columns show the results for drilling with speed of 500 rpm and feed-rate of 35 mm/min

Numerical results of the study are presented in Table 1. In this table, average and tolerance of maximum achieved temperature during each experiment is shown. However, to compare the efficiency of coolants directly, the results are separated for each specific drilling diameter and are also presented in Fig. 2a and Fig. 2b for drilling diameters of 2.7 and 3.5 mm, respectively. In these figures, the combination of each drilling speed and drilling feed-rate is considered as a particular condition in experiments. Thus, the 3 cooling conditions are explicitly compared on the diagrams for each particular combination of the drilling parameters. For example, for drill bit diameter of 3.5 mm, speed of 500 rpm, and feed-rate of 35 mm/min, maximum temperature during experiments for the 3 cooling conditions can be compared on the first column of the diagram in Fig. 2b.

**Table 1:** The numerical results of the entire experiments. In the table, the following codes are used. S1: speed of 500 rpm; S2: speed of 1000 rpm; S3: speed of 1500 rpm; F1: feed-rate of 35 mm/min; F2: feed-rate of 65 mm/min; F3: feed-rate of 85 mm/min. Therefore, for example, S1-F2 means drilling with speed of 500 rpm and feed-rate of 65 mm/min

Drilling diameter	Coolant type	Drilling speed and feed-rate								
		S1-F1	S1-F2	S1-F3	S2-F1	S2-F2	S2-F3	S3-F1	S3-F2	S3-F3
		Maximum temperature of experiment (°C)								
2.7 mm	None	36.0 ± 3	36.0 ± 2	39.0 ± 2	28.9 ± 2	39.0 ± 3	74.0 ± 1	53.0 ± 2	35.2 ± 3	62.0 ± 1
	Saline	35.7 ± 2	32.0 ± 1	36.0 ± 3	28.0 ± 1	27.0 ± 4	44.8 ± 2	33.0 ± 3	29.0 ± 2	30.0 ± 1
	OpSite	27.0 ± 2	25.1 ± 3	33.0 ± 2	25.1 ± 2	25.0 ± 3	35.2 ± 1	22.0 ± 2	22.0 ± 3	21.4 ± 1
3.5 mm	None	36.0 ± 2	36.0 ± 3	38.0 ± 1	45.7 ± 2	43.7 ± 2	65.5 ± 1	66.0 ± 1	42.3 ± 3	41.0 ± 3
	Saline	26.0 ± 1	34.7 ± 2	27.9 ±1	37.2 ± 2	38.9 ± 1	31.0 ± 2	49.7 ± 3	36.2 ± 1	38.4 ± 3
	OpSite	24.0 ± 2	30.0 ± 1	25.7 ±3	34.0 ± 2	37.0 ± 3	29.1 ± 2	31.6 ± 3	30.0 ± 1	26.5 ± 2

In regard to statistical analysis, for normality test, the Shapiro-Wilk test was applied. In this test, null hypothesis states that data are taken from normal distributed population. So, when *P*>0.05, null hypothesis is accepted and data are called as normally distributed. According to this test, our results showed normal distribution for each group of 2.7 mm and 3.5 mm diameters. As a further step, the one-way analysis of variance (ANOVA) was used to determine whether there were any statistically significant differences between the means of the three independent groups. The results regarding the difference between the three cooling methods had *P* value of < 0.05 which indicates the differences are significant. Since ANOVA test only reveals differences between groups and does not examine each group separately, Dunnett T3 test was used to examine group by group. The results of this test also showed the significant differences about using of OpSite spray against normal saline and without cooling condition (Table 2).

**Table 2:** The results of statistical analyses for the entire experiments.

Statistical tests	Coolant type	Drilling diameter	
		2.7 mm	3.5 mm
		*P* value	
Shapiro-Wilk	None	0.07	0.311
	Saline	0.20	0.848
	OpSite	0.07	0.913
**One-Way ANOVA**	All	0.000	0.004
**Dunnett T3**	None	0.052	0.052
	Saline	0.000	0.000
	OpSite	0.045	0.045

## Discussion

There are a variety of studies on investigating the effect of external cooling during drilling of bone and all of them emphasize its positive influence in limiting the maximum temperature elevation [22]. Augustin et al. [8] investigated the effect of external irrigation with water as coolant. They indicated that while there are several parameters that increase bone temperature during drilling, cooling (as external irrigation in their case) can be the only and the most important factor in limiting this increase in bone temperature and must be used for bone drilling. On the other hand, Shakouri et al. [3], Sener et al. [17], Al-Dabag and Sultan [23], and Sindel et al. [24] determined the efficiency of external cooling with normal saline as coolant. They also indicated similar positive influence in limiting the maximum temperature elevation. Therefore, the lower maximum temperatures, which are seen in Fig. 2a, b for external cooling with normal saline in comparison to the condition that there was no cooling, are not a surprise; the results thoroughly support the previous studies on external cooling.

Moreover, studies on the effect of drilling speed and feed-rate on temperature elevation are not consistent [22]. This was also the case when external cooling was employed since in the experiments of Shakouri et al. [3], there is no trend for temperature variations with changes in drilling speed, neither without cooling nor with external cooling. In spite of this, the external cooling efficiency in limiting the temperature rise is observed with all combinations of parameters in the current study.

According to Table 1 and Fig. 2, using OpSite spray not only limited the maximum temperature elevation in our experiments, but also shows much better results in comparison to normal saline. It is more important if consider the lower risks of infection for this coolant since it is not liquid but rather is in the form of spray [3]. Furthermore, according to the literature, for occurrence of thermal osteonecrosis, there is a reverse exponential relationship between thermal necrosis temperature and necrosis time. Whereas the exact threshold temperature for thermal osteonecrosis in human bone is unknown, heat transfer to the bone cells in an average temperature of 47 °C for 1 min is believed to be the threshold [22]. However, OpSite successfully limited the temperature elevation and the highest recorded temperature for cooling with OpSite spray was 37 °C. Thus, the risks of thermal osteonecrosis during drilling and consequent risks of loosening and failure in the implants and orthopedic fixation screws are much lower when using OpSite as coolant.

With regard to the results of this study, it can be concluded that though employment of a cooling system has positive effects in controlling temperature rise during drilling of bone, using OpSite spray as coolant for external cooling is recommended in orthopaedic surgeries due to lower risks of thermal osteonecrosis (better subsequent osseointegration and thus more success rate) and infection.

However, more in vivo studies on its clinical success are needed, both on animals and human. Moreover, to have a better conclusion, further osteonecrosis evaluations on histopathology of the surrounding tissue may be helpful.

## Conclusion

In this study, use of OpSite as coolant in bone drilling was proposed, thus, for three conditions, without cooling, external cooling with normal saline, and external cooling with OpSite spray, the efficiency of OpSite as coolant was studied. In this regard, entire combinations of some drilling parameters, including drill bit diameter, drilling speed, and drilling feed-rate were considered. For the whole experiments, while cooling with normal saline resulted in lower maximum temperatures than without cooling condition, OpSite had even better results and limited the temperature elevation during drilling of bone efficiently. Therefore, due to its lower risks for infection and thermal osteonecrosis in comparison to normal saline, it can be recommended for clinical surgeries in cooling applications.

## Data Availability

All data generated or analyzed during this study are included in this published article.
